# Current News

**Published:** 1904-11

**Authors:** 


### Current News.

### SOUTHERN BRANCH OF THE NATIONAL DENTAL ASSOCIATION.

The eighth annual meeting of the Southern Branch of the National Dental Association will be held February 21 to 23, 1905, at Memphis, Tenn.

J. A. Gorman,
*Corresponding Secretary.*

Asheville, N. C.

### SOUTH DAKOTA STATE BOARD OF DENTAL EXAMINERS.

The South Dakota State Board of Dental Examiners will hold its next regular session for the examination of candidates at Sioux Falls, S. D., Tuesday, December 6, beginning at 1.30 p.m. All candidates will be required to bring operating-instruments, prepared to do all kinds of clinical operative work, also a bridge of not less than four teeth, including one Richmond and one gold shell crown invested ready to solder. All candidates must positively send in their application to G. W. Collins, secretary, Vermilion, S. D., not later than December 2.

G. W. Collins,
*Secretary.*

